# A modified Wald interval for the area under the ROC curve (AUC) in diagnostic case-control studies

**DOI:** 10.1186/1471-2288-14-26

**Published:** 2014-02-19

**Authors:** Martina Kottas, Oliver Kuss, Antonia Zapf

**Affiliations:** 1 Institute for Biostatistics, Hannover Medical School, Carl-Neuberg-Str. 1, 30625 Hannover, Germany; 2 Institute for Biometry and Epidemiology, German Diabetes Center, Leibniz Institute for Diabetes Research at Heinrich Heine University Düsseldorf, Auf’m Hennekamp 65, 40225 Düsseldorf, Germany; 3 Department of Medical Statistics, University Medical Center Göttingen, Humboldtallee 32, 37073 Göttingen, Germany

**Keywords:** AUC, Diagnostic study, Biomarker study, Wald interval

## Abstract

**Background:**

The area under the receiver operating characteristic (ROC) curve, referred to as the AUC, is an appropriate measure for describing the overall accuracy of a diagnostic test or a biomarker in early phase trials without having to choose a threshold. There are many approaches for estimating the confidence interval for the AUC. However, all are relatively complicated to implement. Furthermore, many approaches perform poorly for large AUC values or small sample sizes.

**Methods:**

The AUC is actually a probability. So we propose a modified Wald interval for a single proportion, which can be calculated on a pocket calculator. We performed a simulation study to compare this modified Wald interval (without and with continuity correction) with other intervals regarding coverage probability and statistical power.

**Results:**

The main result is that the proposed modified Wald intervals maintain and exploit the type I error much better than the intervals of Agresti-Coull, Wilson, and Clopper-Pearson. The interval suggested by Bamber, the Mann-Whitney interval without transformation and also the interval of the binormal AUC are very liberal. For small sample sizes the Wald interval with continuity has a comparable coverage probability as the LT interval and higher power. For large sample sizes the results of the LT interval and of the Wald interval without continuity correction are comparable.

**Conclusions:**

If individual patient data is not available, but only the estimated AUC and the total sample size, the modified Wald intervals can be recommended as confidence intervals for the AUC. For small sample sizes the continuity correction should be used.

## Background

The result of a diagnostic test is in general not binary (positive/negative) but a quantitative parameter (such as a biomarker). If an appropriate threshold for the quantitative parameter has not yet been defined, the receiver operating characteristic (ROC) curve and in particular the area under this curve, are appropriate for evaluating the overall accuracy of the diagnostic test [1]. The ROC curve is a plot of sensitivity (true positive rate) and one minus specificity (true negative rate) for each possible threshold value of the biomarker of interest. In the case of complete separation of cases and controls, the area under the ROC curve (AUC) is equal to one. For a diagnostic test, which is no better than chance, the AUC is 0.5. In early phase diagnostic studies, amongst others, the aim is in general to get a first impression of the overall diagnostic accuracy.

Early phase diagnostic studies often exhibit three characteristics: 

1. The sample sizes are small. For example, in the systematic review by Cochrane and Ebmeier of diffusion tensor imaging (DTI) as a candidate biomarker for the diagnosis of Parkinson disease, the median total sample size of the 21 selected studies was 32 (mean = 39) [2]. The largest study in the systematic review by Wang et al. of cardiac testing for coronary artery disease in potential kidney transplant recipients included 219 patients [3].

2. A case-control study design with comparable sizes in the two groups is chosen [1,4] (i.e. case-control ratio ≈1:1). Controls are generally healthy volunteers, patients with benign disease, or patients with a disease within the scope of the differential diagnosis (see for example [5-7]).

3. Diagnostic tests or biomarkers yield large values for the AUC. For example in the systematic review by Wang et al. the AUC’s of the different diagnostic tests were between 0.78 and 0.91 [3].

Many different confidence intervals have been proposed for the AUC. Bamber suggested in 1975 a variance estimator and corresponding confidence interval for the AUC, which was the starting point for many authors [8]. Qin and Hotilovac compared in 2008 nine nonparametric intervals [9]. Their conclusion was that the empirical likelihood-based interval and the Mann-Whitney interval with Logit transformation lead to good coverage accuracy. The Mann-Whitney interval without transformation was not recommended by the authors, however, it is used in the ROC statement of PROC LOGISTIC in SAS. A parametric approach is the AUC under the binormal ROC curve (see for example the book of Pepe [10]).

But because all confidence intervals for the AUC are relatively complicated to implement, and some of them either do not maintain or do not exploit the type I error probability *α* for small sample sizes or large values for the AUC, we investigated alternatives. Our basic approach was to use simple two-sided confidence intervals for a single proportion, because the AUC can be interpreted as a probability (that a randomly chosen diseased individual has a larger value for the biomarker than a randomly chosen non-diseased individual, see for example [11], formula (1.3)). The simplest confidence interval is the Wald interval, which tends to yield anti-conservative results [12]. As an alternative we propose a conservative version with a modified variance estimator, based on Bamber [8]. Newcombe compared seven confidence intervals for a single proportion and recommended the Wilson interval ("Of the methods that perform well, only the score method is calculator friendly.") [13]. Wilson’s score interval is still suggested, particularly for proportions close to 0 or 1 (see for example the article of He et al. [14]). Agresti and Coull recommended a modified Wald interval, which has similar behaviour to the Wilson interval for a two-sided type I error of 5%, but a simpler formula [15]. The Clopper-Pearson interval is another alternative. It is an exact interval but tends to yield conservative results.

In this article we compare the modified, conservative Wald confidence interval (with and without continuity correction) with the Mann-Whitney interval with Logit transformation interval as main reference. Furthermore Bamber’s interval, the Mann-Whitney interval without transformation, and the binormal AUC are included. For the family of intervals for a single proportion Wilson’s score interval (with and without continuity correction), the Agresti-Coull interval, and the Clopper-Pearson interval are added. In line with the recommendations of Burton et al. [16] we compare the intervals in terms of coverage probability, interval length, and statistical power. The aim of this article is to determine if one of the intervals is an appropriate alternative to the Mann-Whitny interval with Logit transformation; and if so, in which situations it performs well. In the next section we describe the statistical model and the different confidence intervals. Then the results of the simulation study and of an example are presented. Finally, the results are summarised and discussed, and recommendations are given.

## Methods

Given the case-control design, suppose there are *n*_1_ cases and *n*_0_ controls. This gives a total sample size of *n* = *n*_1_ + *n*_0_. Each individual observation *X*_*is*_(*s* = 1,…,*n*_*i*_) in group *i* = 0,1 follows the normalized version of the marginal distribution function *F*_*i*_(*x*) = *P*(*X*_*is*_ < *x*) + 0.5 · *P*(*X*_*is*_ = *x*) (for details refer to [11,17]). As an unbiased point estimator for the AUC (which is inserted in all intervals but in the binormal interval) the numerator of the Mann-Whitney test statistic was used (for details and proof see Brunner et al. [18], (3.2) and Bamber [8]). To achieve this, all *n* observations are ranked, and for each status group *i* = 0,1 the average rank ${\bar{R}}_{i.}$ is calculated. Then the estimated AUC is

(1) $$\hat{\text{AUC}}=\frac{1}{n}\left({\bar{R}}_{1.}-{\bar{R}}_{0.}\right)+0.5\;.$$

### Confidence intervals for the AUC

In the following the confidence intervals for the AUC, which are used for comparison in the simulation study, are shortly described. The notation needed for the formulas is given in Table 1, and the formulas themselves are given in Table 2. Qin and Hotilovac determined that the Mann-Whitney interval with Logit transformation (in the following abbreviated as LT) has good coverage probability [9], therefore the LT interval serves as main reference here. The advantage of the LT interval is that it is always range-preserving (that is, both confidence limits lie within the interval [0,1]), but the disadvantage is that it cannot be calculated if the estimated AUC is equal to one. We also included the Mann-Whitney interval without transformation (M-W), which was also investigated by Qin and Hotilovac. They stated that the M-W interval "... suffers from low coverage accuracy for high values of AUC...", but it is often used in statistical programs (for example in the ROC statement of PROC LOGISTIC in SAS, where it is referred to as a Wald interval [19]). The M-W interval can also not be calculated for an AUC equal to 1, and is not range preserving (in SAS the upper limit of the interval is set to 1 if it is greater than 1). Many approaches are based on the confidence interval suggested by Bamber [8], and it is also the starting point for our modified Wald interval. Therefore we implemented Bamber’s interval (denoted Bamber) as another reference method. Bamber’s interval is also not range-preserving.

**Table 1:** **Needed notation for the formulas of the different confidence intervals in Table**2

**Notation**	**Explanation**
$\text{logit}(\text{AUC})=\text{log}\left(\frac{\text{AUC}}{1-\text{AUC}}\right)$	Logit transformation of the AUC
$\text{expit}(\cdot )=\frac{\text{exp}(\cdot )}{\text{exp}(\cdot )+1}$	Back transformation of the Logittransformation
*z* = *z*_1-*α*/2_	1-*α*/2 quantile of the normal distribution
*s*	Empirical standard deviation of AUC
*se*	Standard error of AUC by Bamber [8]
${\text{AUC}}^{*}=\Phi \left(\frac{({\bar{X}}_{1}-{\bar{X}}_{0})/s_{1}}{\sqrt{1+{\left(s_{0}/s_{1}\right)}^{2}}}\right)$	AUC for the binormal ROC curve (*s*_*i*_,*i* = 0,1,as empirical estimator of *σ*_*i*_)
*s*^∗^	Empirical standard deviation of *AUC*^∗^
$t=\frac{z_{1-\alpha /2}^{2}}{n}$	Factor for the Wilson interval
$\tilde{\text{AUC}}=\frac{\hat{\text{AUC}}\cdot n+2}{n+4}$	Modified AUC (for the A-C interval)
$k=\text{round}(\hat{\text{AUC}}\cdot n)$	Estimated number of successes (for the C-Pinterval)
*f*(1 - *α*/2,*df*1,*df*2)	1-*α*/2 quantile of the F distribution with *df*1 and *df*2 degrees of freedom

**Table 2:** Formulas for the different confidence intervals from section Methods

**Confidence interval (denotation)**	**Range-preserving**	**Result for AUC = 1**	**Limits**
Logit-transformation-based (LT)	Yes	No	$\text{expit}\left(\text{logit}(\hat{\text{AUC}})\pm z\cdot s/\left(\hat{\text{AUC}}(1-\hat{\text{AUC}})\sqrt{n}\right)\right)$
Mann-Whitney (MW)	No	No	$\hat{\text{AUC}}\pm z\cdot s/\sqrt{n}$
Bamber (Bamber)	No	Yes	$\hat{\text{AUC}}\pm z\cdot \text{se}$
Binormal (Binormal)	No	No	$\text{AU}C^{*}\pm z\cdot s^{*}/\sqrt{n}$
Wilson (Wilson)	Yes	Yes	$\left(\hat{\text{AUC}}+0.5t\right)/\left(1+t\right)\pm \sqrt{\hat{\text{AUC}}(1-\hat{\text{AUC}})t+0.25t^{2}}/\left(1+t\right)$
... with continuity correction (Wilson-cc)	Yes	Yes	lower: $\left(2n\hat{\text{AUC}}+z^{2}-1-z\sqrt{z^{2}-2-1/n+4\hat{\text{AUC}}(n(1-\hat{\text{AUC}})+1)}\right)/$ (2(*n* + *z*^2^))
			upper: $\left(2n\hat{\text{AUC}}+z^{2}+1+z\sqrt{z^{2}+2-1/n+4\hat{\text{AUC}}(n(1-\hat{\text{AUC}})\;+\;1)}\right)/$ (2(*n* + *z*^2^))
Agresti-Coull (A-C)	No	No	$\tilde{\text{AUC}}\pm z\sqrt{\frac{\tilde{\text{AUC}}(1-\tilde{\text{AUC}})}{n+4}}$
Clopper-Pearson (C-P)	Yes	No	lower: (*k* · *f*(*α*/2,2*k*,2(*n* - *k* + 1)))/ (*n* - *k* + 1 + *k* · *f*(*α*/2,2*k*,2(*n* - *k* + 1)))
			upper: ((*k* + 1)*f*(1 - *α*/2,2(*k* + 1),2(*n* - *k*)))/ (*n* - *k* + (*k* + 1)*f*(1 - *α*/2,2(*k* + 1),2(*n* - *k*)))
Modified Wald (Wald)	No	No	$\hat{\text{AUC}}\pm z\sqrt{\frac{\hat{\text{AUC}}(1-\hat{\text{AUC}})}{0.75n-1}}$
... with continuity correction(Wald-cc)	No	Yes	$\hat{\text{AUC}}\pm z\sqrt{\frac{\hat{\text{AUC}}(1-\hat{\text{AUC}})}{0.75n-1}}+1/(2n)$

A parametric approach is the binormal ROC curve (denoted Binormal), assuming normal distributions for the test results of the cases and of the controls ( $X_{1}∼N(\mu_{1},\sigma_{1}^{2}),X_{0}∼N(\mu_{0},\sigma_{0}^{2})$). The corresponding area under the resulting curve is called binormal AUC. The binormal AUC is estimated using the empirical estimators of the distribution functions (for formula see Table 2, for details see for example the book of Pepe [10]).

### Confidence intervals for a single proportion

The Wilson score interval [20] and the Wilson interval with continuity correction (denoted Wilson and Wilson-cc) are known for their good properties in the case of proportions near to 0 or 1 [14]. The formulas are more complicated than the Wald interval, but only the quantile, the total sample size *n*, the point estimator $\hat{\text{AUC}}$ and constants are needed (see Table 2). The intervals can also be calculated in the case of AUC equal to 1, and the limits are always range-preserving. The 95% interval of Agresti and Coull [15] (denoted A-C) as a Wald interval adding two "successes" and two "failures" has a similar behaviour as the Wilson interval, but a simpler formula (see Table 2). In the usual setting in which it is applied, the exact confidence interval of Clopper and Pearson [21] (denoted C-P) maintains type I error by definition. However, this property is not valid here because the AUC is a probability relating two independent groups rather than to a group and a subgroup. The interval can be calculated with a finite formula (see for example the article of Agresti and Coull [15]). In the case of $k=\text{round}(\hat{\text{AUC}}\cdot n)=n$ the interval cannot be calculated. The corresponding formulas for all intervals are given in Table 2.

### Modified Wald intervals

The Wald confidence interval is very easy to calculate and in general has good properties. But it is known that for small sample sizes it becomes anti-conservative [12]. Therefore we propose a Wald interval with a modified variance estimator. In his article Bamber gave beside the estimator for the variance (denoted Bamber interval, see above) also the maximum variance for the case of continuous **X**_0_ and **X**_1_ with monotonic posterior (= the larger the measured value, the larger the probability for the presence of the disease). According to Bamber the estimated asymptotic maximum variance is ${\hat{\sigma }}_{\text{max}}^{2}=\frac{\hat{\text{AUC}}\cdot (1-\hat{\text{AUC}})}{0.75\cdot n-1}$ (for balanced sample sizes, derived from [8,22]).

The formulas for the corresponding Wald intervals with and without continuity correction (denoted Wald and Wald-cc) are given in Table 2. One advantage of the Wald interval with continuity correction is that it can also be calculated for an estimated AUC equal to 1. The upper and the lower limit of the Wald interval without continuity correction would be equal to 1 for AUC = 1. The Wald intervals are not range preserving.

### Simulation methods

The simulation program was implemented in SAS/IML and 10 000 simulation runs were used. The binormal intervals were calculated only for the first 1 000 simulation runs, because of it’s high computation time. First we generated normally distributed data, independently for the two groups, with *μ* = 0 and $\sigma_{0}^{2}=\sigma_{1}^{2}=1$ as the variances of the controls and the cases, respectively. Then the values for the cases were shifted by $\Phi^{-1}({\text{AUC}}_{0})\cdot \sqrt{\sigma_{0}^{2}+\sigma_{1}^{2}}$ to obtain the true AUC (*AUC*_0_).

In the simulation study we varied the true AUC, the sample size, the case-control ratio, the measurement scale and the variance of the cases (for details see Table 3). The true AUC ranged from 0.7 to 0.9 (upper limit corresponding to the systematic review of Wang et al. [3]). Because of the spike shape of the type I error for increasing *AUC*_0_ (see for example Agresti and Coull [15]), we simulated for each *AUC*_0_ also *AUC*_0_ ± 0.01 and calculated the mean coverage probability and interval length of the three scenarios. The total sample size *n* ranged from 40 to 200 (corresponding to the systematic reviews of Cochrane and Ebmeier [2] and of Wang et al. [3]). For the main analysis identical sample sizes were used for the two groups, *n*_1_ = *n*_0_ = *n*/2 (i.e. the case-control ratio is 1:1). Following the recommendations of Burton et al. [16] we investigated the two-sided coverage probability (theoretical *α* set to 5% two-sided), the interval length, and the statistical power. Regarding the interval length we set for the confidence intervals, which are not range-preserving, the upper limit to 1 if it was greater than 1. Furthermore, we investigated the robustness with respect to unbalanced sample sizes (changing case-control ratio), skewed distributions, variance heterogeneity and categorical outcomes.

**Table 3:** Varied factors in the simulation study

**Factor**	**Variation**	**Results in paragraph**
True AUC (*AUC*_0_)	0.7,0.8,0.9 (each ±0.01)	
Sample size (*n*)	40,100,200	
Case-control ratio	1:1	Interval length, coverage probability
Measurement scale	Continuous	
Variance of the cases (*σ*_1_), *σ*_0_ = 1	1	
Case-control ratio	1:2, 1:9	
Measurement scale	Ordinal with five categories	Robustness evaluation
Variance of the cases (*σ*_1_), *σ*_0_ = 1	0.5,2,3	
AUC under the alternative hypothesis (*AUC*_1_)	0.700,0.701,…,0.85;	Statistical power
	0.800,0.801,…,0.99	

The SAS simulation program and all results as tables are given in the Additional files 1 and 2.

## Results and discussion

We first simulated data for the nine combinations of *AUC*_0_ and total sample size *n* (with 1:1 case-control ratio). Under specific conditions the LT and M-W intervals ( $\hat{\text{AUC}}=1$) and the C-P interval ( $k=\text{round}(\hat{\text{AUC}}\cdot n)=n$) cannot be computed (see Methods). The LT interval could not be computed only for the combination of small sample size and high AUC (*n* = 40, *AUC*_0_ = 0.9) and only for 14 of the 10 000 simulation runs. For the same scenario the C-P could not be computed for 125 simulation runs. The C-P interval could also not be computed for *n* = 40 and *AUC*_0_ = 0.8 for two simulation runs.

### Interval length

For interval length, across the nine scenarios the Wald intervals tend to be the widest, while the A-C and the Wilson interval tend to be the narrowest. A box plot of the length of the different intervals is given in the Additional file 3: Figure S1. The simulation runs which did not yield intervals (141 runs overall, see above) were ignored.

### Coverage probability

**Figure 1:**
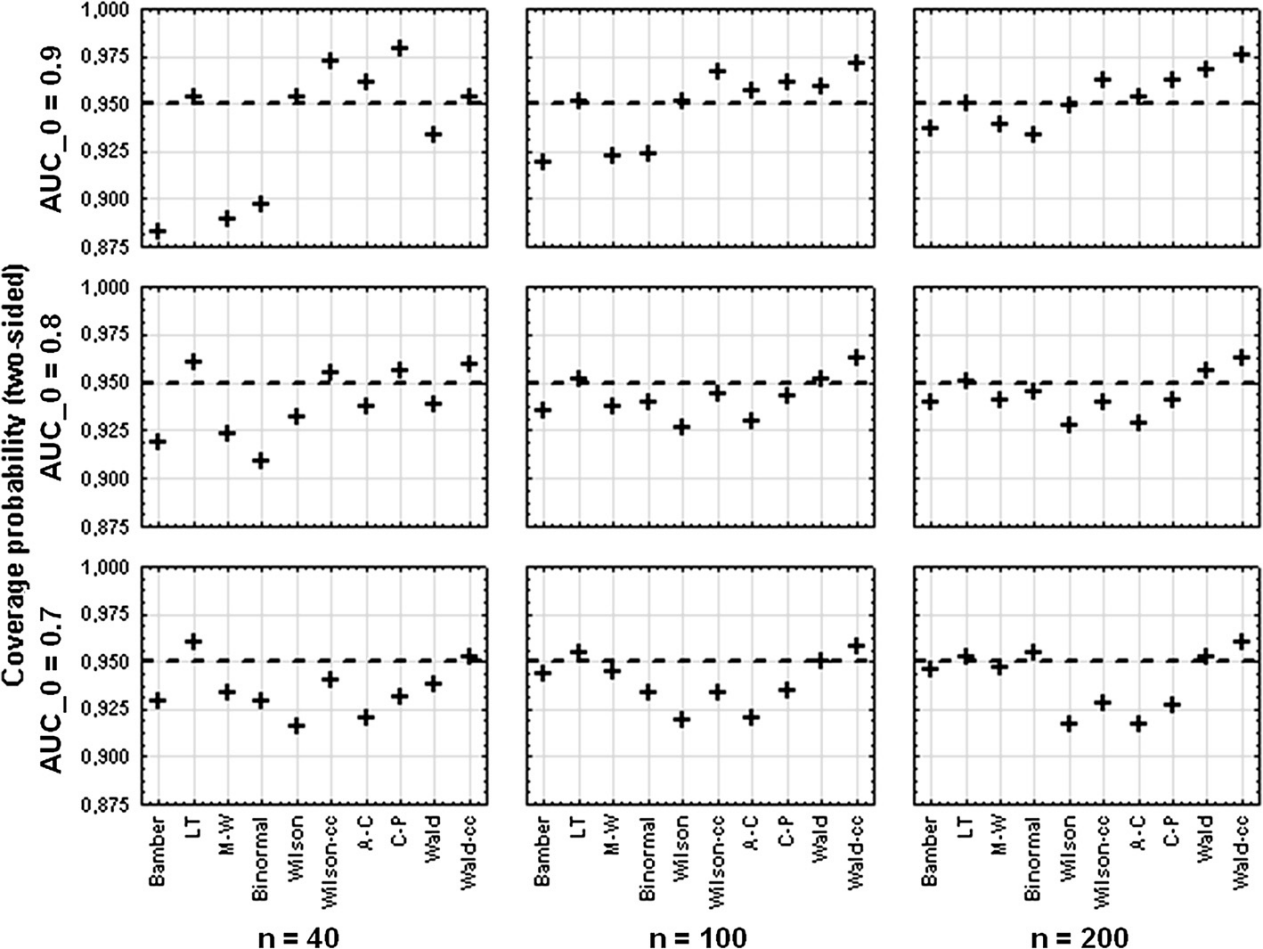
**Effect of varying AUC and sample size.** Dot plot for the coverage probability of the different intervals for varying AUC and sample size.

The coverage probability for the nine scenarios is shown as dot plot dependent on *AUC*_0_ and *n* in Figure 1. The coverage probability of the LT interval is slightly conservative for small sample sizes (coverage probability up to 96%), but for a sample size of 100 or 200 the coverage is independent of the true *AUC*_0_ and equals roughly 95%. The M-W, the Binormal, and Bamber’s interval have a coverage probability of about 95% only for a sample size of 200 and a true AUC of 0.7. In all other cases the intervals are quite liberal, getting worse with increasing *AUC*_0_ and with decreasing sample size. For *n* = 40 and *AUC*_0_ = 0.9 the coverage probabilities are only 88% for Bamber, 89% for M-W, and 90% for Binormal.

The Wilson interval without continuity correction has a coverage probability of nearly 95% for an *AUC*_0_ of 0.9, independent of the sample size. But for lower *AUC*_0_‘s the coverage of the Wilson interval drops to 92%. The Agresti-Coull interval, the Wilson interval with continuity correction, and the Clopper-Pearson interval tend to be liberal for an *AUC*_0_ of 0.7 (coverage between 92% and 94%), and become quite conservative for higher AUC’s (coverage up to 98%).

The modified Wald interval without continuity correction is liberal for small sample sizes (93*%*-94*%* coverage), for larger sample sizes the coverage is comparable to the LT interval. However, for a large sample size of *n* = 200 and a high *AUC*_0_ of 0.9 the Wald interval becomes conservative (coverage of 97%). The coverage probability of the continuity corrected Wald interval is very similar to the LT interval, but for larger sample sizes the Wald-cc interval becomes conservative (coverage up to 98%).

Because overall the LT and the Wald intervals maintained the type I error best, we restricted subsequent investigations to these three intervals.

### Statistical power

**Figure 2:**
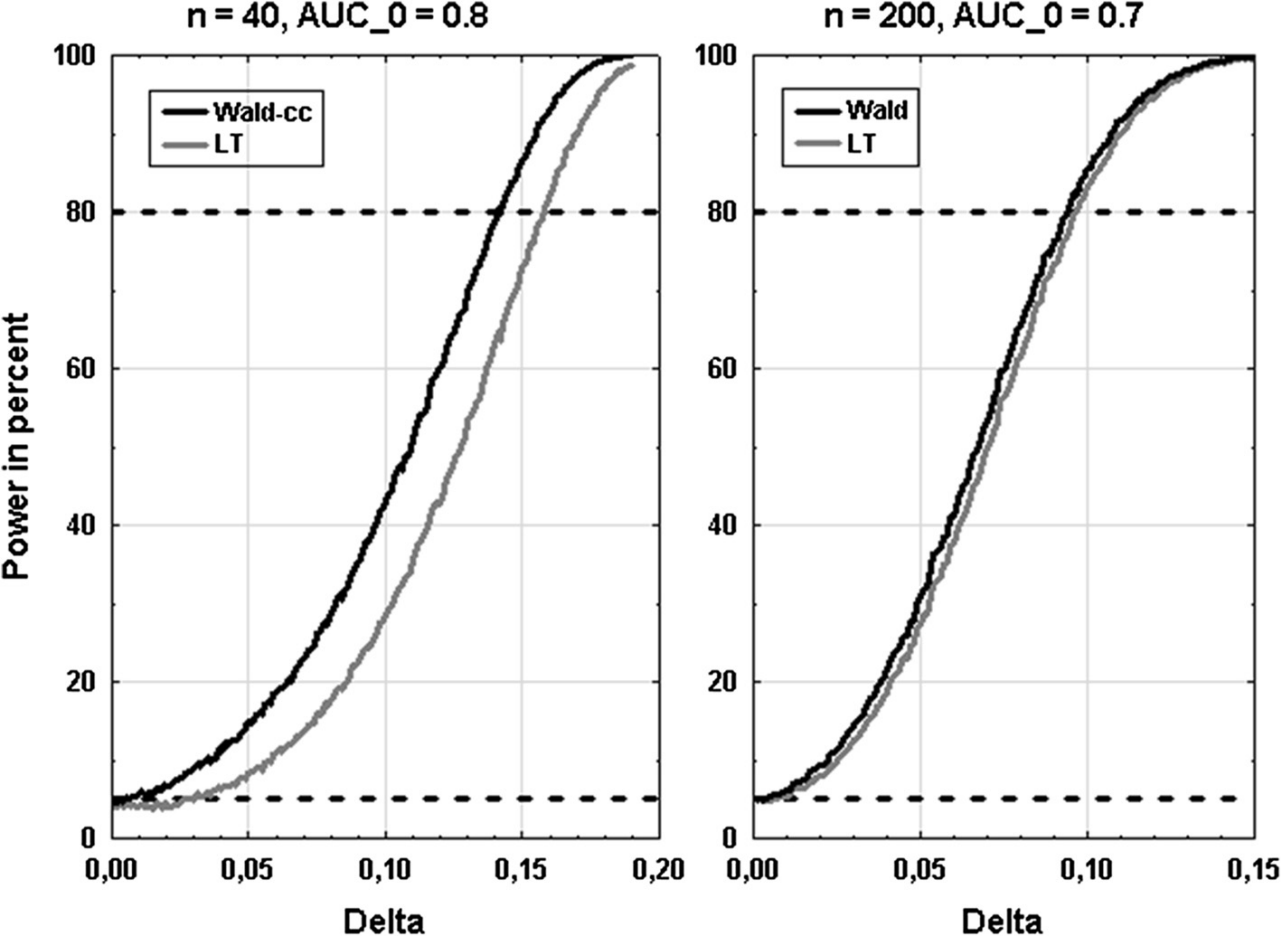
**Power curves for two scenarios.** Power curves for the LT and the Wald-cc interval for a 1:1 case-control ratio, *n* = 40, and *AUC*_0_ = 0.8 (left side), and for the LT and the Wald interval for a 1:1 case-control ratio, *n* = 200, and *AUC*_0_ = 0.7 (right side). Delta is the difference between *AUC*_0_ (0.8 respectively 0.7) and *AUC*_1_ (from *AUC*_0_ to 0.99 respectively 0.85).

We compared the power of the LT and the Wald intervals for two scenarios, where the intervals yielded comparable coverage probabilities. This means that the LT and the Wald interval were compared for *n* = 200 and *AUC*_0_ = 0.7, and the LT and the Wald-cc interval for *n* = 40 and *AUC*_0_ = 0.8. The corresponding power curves are presented in Figure 2. While the Wald interval has only marginal higher power than the LT interval (right side of Figure 2, maximum difference of 4%), the Wald-cc interval has much more power than the LT interval (left side of Figure 2, maximum difference of 17%).

### Robustness evaluation

**Figure 3:**
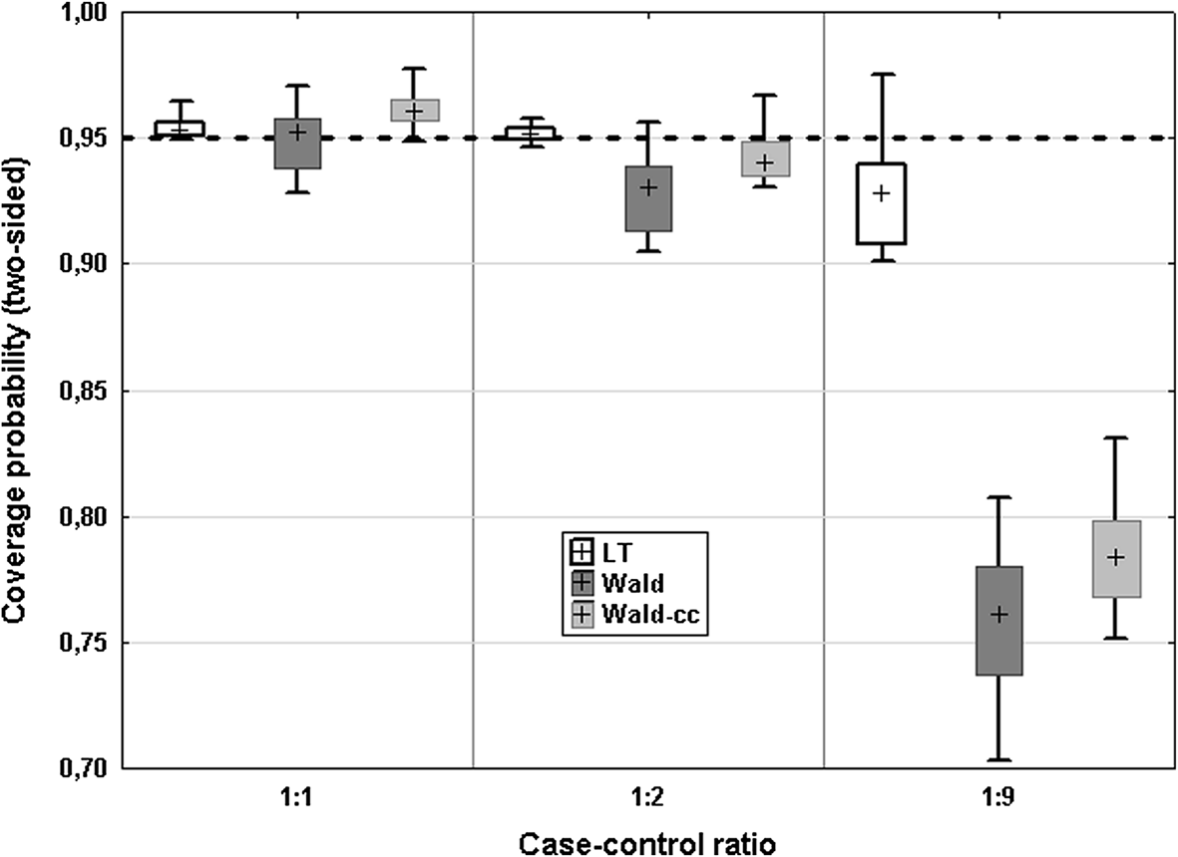
**Effect of varying case-control ratio.** Box plot of the coverage probability for varying case-control ratios (*n* = (40,100,200) and *AUC*_0_ = (0.7,0.8,0.9), cross = median, box = 25*%*-75*%* quantile, whiskers = min - max).

We first investigated the robustness to unbalanced sample sizes. The case-control ratio ranges from 1:1 to 1:9 (in the article of Cochrane and Ebmeier [2] the maximum ratio was 1:2). The results are presented in Figure 3. With increasing imbalance, the coverage probability becomes lower for the Wald intervals as well as for the LT interval. However, the LT has still a median coverage probability of 95% for a case-control ratio of 1:2, and becomes slightly liberal for a case-control ratio of 1:9. In contrast the Wald intervals are slightly liberal even for a case-control ratio of 1:2 (median coverage probability of 93% for the Wald and 94% for the Wald-cc interval), and become very liberal for a case-control ratio of 1:9 (median coverage 76% respectively 78%).

The LT and the Wald intervals are robust with respect to non-normal distributions, which is important because biomarker follow often a skewed distribution. This is not surprising, because the numerator of the Mann-Whitney test statistic as point estimator is based on the ranks of the measurements. Therefore the estimators and accordingly the LT interval are invariant under any monotone transformation. The Wald-interval is based only on the point estimator and the sample size. Thus the LT and the Wald intervals are robust with respect to non-normal distributions.

Because test results can also be ordinal (especially in studies involving imaging techniques), we investigated the coverage probability after categorizing the normally distributed data into five categories (using the percentiles 20, 40, 60, and 80). For continuous data, the median coverage probability of the LT and of the Wald interval is about 95%, while the median coverage of the Wald-cc interval is 96%, and the range of the LT interval is smaller than the range of the Wald intervals. For ordinal data the median coverage probability of the LT interval increases only from 95.3*%* to 95.4*%*, but the range becomes as large as the range of the Wald intervals. The median coverage of the Wald intervals increases from 95.3*%* to 95.6*%* for Wald, and from 96.1*%* to 96.6*%* for Wald-cc, while the range does not change much. The corresponding figure is given in the Additional file 4: Figure S2.

**Figure 4:**
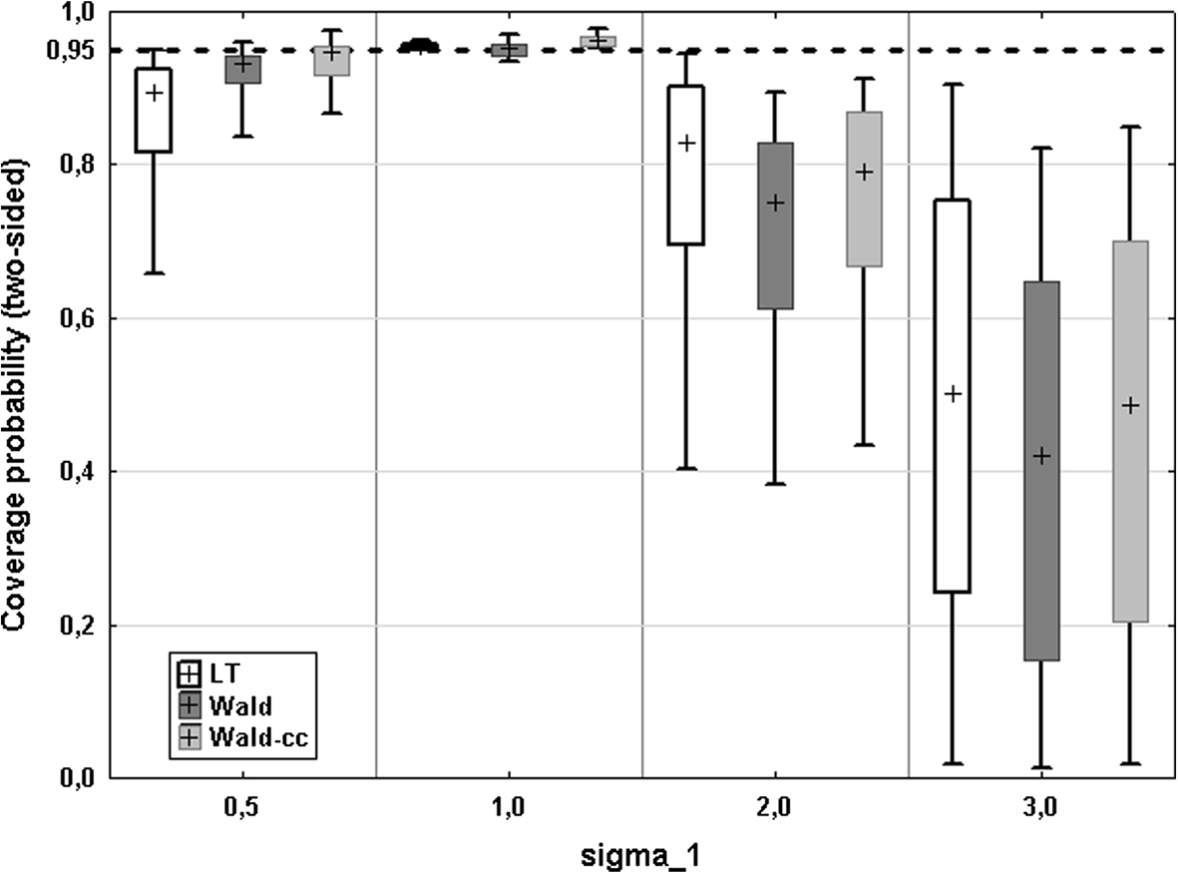
**Effect of variance heterogeneity.** Box plot of the coverage probability for increasing variance of the cases *σ*_1_ (variance of the controls *σ*_0_ = 1,*n* = (40,100,200) and *AUC*_0_ = (0.7,0.8,0.9), cross = median, box = 25*%*-75*%* quantile, whiskers = min - max).

To investigate the robustness regarding variance heterogeneity we generated data for *n* = (40,100,200) with a 1:1 case-control ratio and *AUC*_0_ = (0.7,0.8,0.9) (same nine scenarios as above). The variance of the control group (*σ*_0_) was set to 1, while the variance of the cases (*σ*_1_) was set to 0.5, 1 (homogeneity), 2, or 3. The median coverage probability of the Wald intervals does not change much for *σ*_1_ = 0.5, while the median coverage of the LT interval decreases to 89%. For variance heterogeneity in the other direction all three intervals (LT, Wald, Wald-cc) become very liberal with increasing *σ*_1_, which can be seen in Figure 4. Regarding the interval length, all three intervals become broader for *σ*_1_ = 0.5, and narrower for increasing *σ*_1_. However, the differences are rather small, and for all intervals comparable (see Additional file 5: Figure S3).

## Example

For illustration we use the example of diagnostic accuracy of CA-19-9 for the diagnosis of pancreatic cancer that was used in the methodical literature before [9,10,23]. The sample sizes in this study were *n*_0_ = 51 and *n*_1_ = 90 (i.e. the case-control ratio was 1:0.6) and the estimated AUC was $\hat{\text{AUC}}=0.86$. Using just this information, all intervals except the LT, the M-W and the binormal interval can be calculated. Using the individual data the LT and the M-W interval can be calculated (dataset taken from http://labs.fhcrc.org/pepe/dabs/datasets.html). The binormal interval cannot be calculated with SAS 9.3 because the approximation leads to negative eigenvalues. The data and the SAS-program for the analysis are given in the Additional files 6 and 7. The results for all methods are very similar (see Table 4). The maximum deviation from the LT interval for the lower limit is 0.012 (for the M-W interval). The Bamber interval is the widest, while the Wilson interval is the narrowest. However, the difference between these two interval lengths is only 0.035.

**Table 4:** **Results of the example from section Example**

**Interval**	**Lower limit**	**Upper limit**	**Interval length**
LT	0.790	0.911	0.121
M-W	0.802	0.921	0.120
Bamber	0.787	0.936	0.149
Wilson	0.795	0.909	0.114
Wilson-cc	0.791	0.912	0.121
A-C	0.794	0.910	0.116
C-P	0.790	0.911	0.122
Wald	0.795	0.928	0.132
Wald-cc	0.792	0.931	0.139

The Binormal interval cannot be calculated because of violated conditions (see Section Example).

## Conclusion

The aim of this article was to investigate whether a modified Wald interval (with or without continuity correction), which is quite easy to implement, is an alternative for the Mann-Whitney interval with logit transformation (LT) for use as a confidence interval for the AUC in diagnostic studies. The simulation study shows that for small sample sizes (here *n* = 40) the Wald interval with continuity correction is as good as the LT interval regarding the coverage probability, and has much more power than the LT interval. For large sample sizes (here *n* = 100,200) the Wald interval without continuity correction is comparable to the LT interval regarding the coverage probability for an *AUC*_0_ up to 0.8, and has slightly more power. For an *AUC*_0_ of 0.9 the Wald interval becomes slightly conservative. The LT interval as well as the Wald intervals are robust to unimodal departures from normality. However, while the LT-interval is quite robust to unbalanced smple sizes and also applicable for ordinal data, the Wald intervals cannot be recommended for very unbalanced or ordinal data. Neither the Wald intervals nor the LT interval are robust to variance heterogeneity.

The other intervals investigated (Mann-Whitney, Bamber, Binormal, Wilson, Wilson with continuity correction, Agresti-Coull, and Clopper-Pearson) cannot be recommended. In particular, the Mann-Whitney interval, which is used in the ROC statement of the PROC LOGISTIC in SAS (referred to there as a Wald interval), Bamber’s interval and the interval for the binormal AUC are much too liberal. This is especially disappointing with respect to the binormal AUC interval, because this one was the only parametric interval under study and had the advantage that the simulation data were generated under its true underlying normal model.

For rather balanced (ratio 1:1 to 1:2) diagnostic case-control studies (which are suitable for proof-of-concept and phase II studies according to the European guideline [1]) the modified Wald intervals are a reasonable alternative to the LT interval. For studies with small sample sizes (about 50 overall) we would recommend to use the Wald interval with continuity correction, for studies with large sample sizes (*n* ≥ 100) we would recommend the Wald interval without continuity correction.

Moreover it is an advantage of the Wald intervals that, in general, they can be computed from published data (only point estimator and total sample size is needed) while the LT interval needs individual patient data for the computation.

## Competing interests

The authors declare that they have no competing interests.

## Authors’ contributions

AZ, MK, OK implemented the confidence intervals. AZ, MK performed the simulation study and wrote the article. OK revised the manuscript. All authors read and approved the final manuscript.

## Pre-publication history

The pre-publication history for this paper can be accessed here:

http://www.biomedcentral.com/1471-2288/14/26/prepub

## Supplementary Material

Additional file 1 **SAS Simulation Program.** SAS simulation program (written in SAS 9.2). Click here for file

Additional file 2 **Result Tables.** All simulation results in separate spreadsheets. Click here for file

Additional file 3 **Figure S1.** Box plot of the interval length for *n* = (40,100,200) with a 1:1 case-control ratio and *AUC*_0_ = (0.7,0.8,0.9) (cross = median, box = 25*%*-75*%*, whiskers = min - max). Click here for file

Additional file 4 **Figure S2.** Box plot of the coverage probability for continuous data and for ordinal data with five categories (*n* = (40,100,200) and *AUC*_0_ = (0.7,0.8,0.9), cross = median, box = 25*%*-75*%*, whiskers = min - max). Click here for file

Additional file 5 **Figure S3.** Box plot of the interval length for increasing variance of the cases *σ*_1_ (variance of the controls *σ*_0_ = 1,*n*=(40,100,200) and *AUC*_0_ = (0.7,0.8,0.9), cross = median, box = 25*%*-75*%* quantile, whiskers = min - max). Click here for file

Additional file 6 **Pancrea.** Data set used as example. Click here for file

Additional file 7 **Pancrea SAS code.** SAS program for analysis of the Pancrea data set. Click here for file
