# Supplementary figures and images for: A modified Wald interval for the area under the ROC curve (AUC) in diagnostic case-control studies
Source: BMC Med Res Methodol. 2014 Feb 19;14:26. doi: 10.1186/1471-2288-14-26 (PMC3938139; doi:10.1186/1471-2288-14-26)

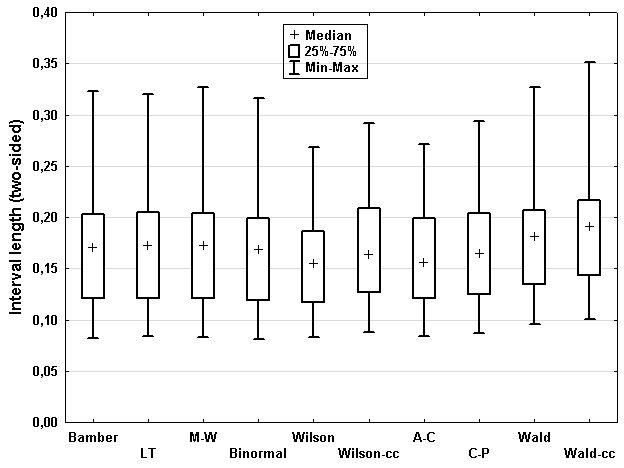

Supplement: Additional file 3 — Figure S1. Box plot of the interval length for n = (40,100,200) with a 1:1 case-control ratio and AUC0 = (0.7,0.8,0.9) (cross = median, box = 25%-75%, whiskers = min - max). [file 1471-2288-14-26-S3.tiff]

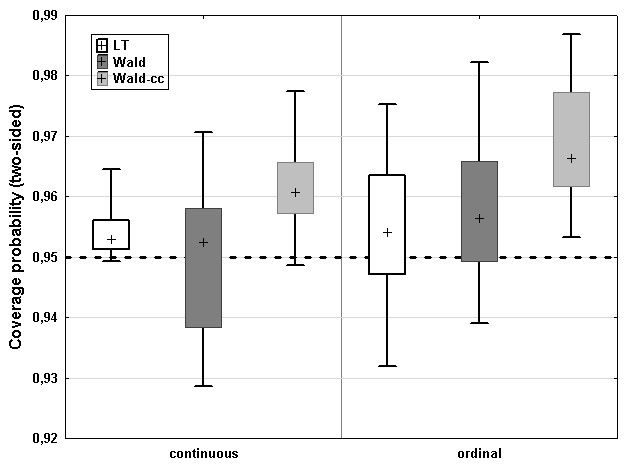

Supplement: Additional file 4 — Figure S2. Box plot of the coverage probability for continuous data and for ordinal data with five categories (n = (40,100,200) and AUC0 = (0.7,0.8,0.9), cross = median, box = 25%-75%, whiskers = min - max). [file 1471-2288-14-26-S4.tiff]

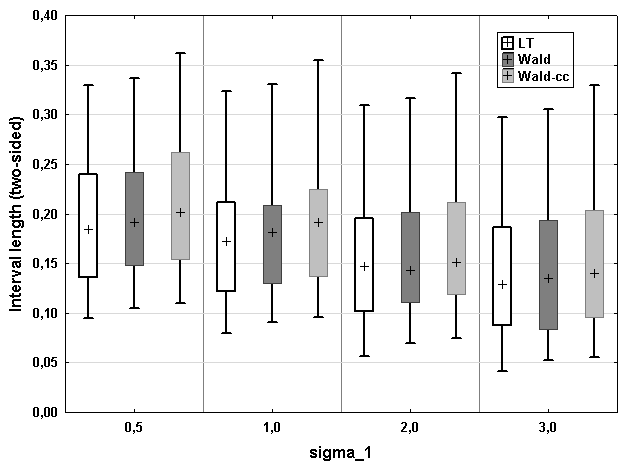

Supplement: Additional file 5 — Figure S3. Box plot of the interval length for increasing variance of the cases σ1 (variance of the controls σ0 = 1,n=(40,100,200) and AUC0 = (0.7,0.8,0.9), cross = median, box = 25%-75% quantile, whiskers = min - max). [file 1471-2288-14-26-S5.tiff]
